# Economic analysis of dengue prevention and case management in the Maldives

**DOI:** 10.1371/journal.pntd.0006796

**Published:** 2018-09-27

**Authors:** Mathieu Bangert, Aishath Thimna Latheef, Shushil Dev Pant, Ibrahim Nishan Ahmed, Sana Saleem, Fathimath Nazla Rafeeq, Moomina Abdulla, Fathimath Shamah, Ahmed Jamsheed Mohamed, Christopher Fitzpatrick, Raman Velayudhan, Donald S. Shepard

**Affiliations:** 1 Department of Control of Neglected Tropical Diseases, World Health Organization, Geneva, Switzerland; 2 World Health Organization Country Office, Malé, Republic of Maldives; 3 Health Protection Agency, Ministry of Health, Malé, Republic of Maldives; 4 Policy Planning and International Health, Ministry of Health, Malé, Republic of Maldives; 5 Department of Control of Neglected Tropical Diseases, World Health Organization Regional Office for South East Asia, New Delhi, India; 6 Schneider Institutes for Health Policy, Heller School for Social Policy and Management, Brandeis University, Waltham, Massachusetts, United States of America; University of Heidelberg, GERMANY

## Abstract

As tourism is the mainstay of the Maldives’ economy, this country recognizes the importance of controlling mosquito-borne diseases in an environmentally responsible manner. This study sought to estimate the economic costs of dengue in this Small Island Developing State of 417,492 residents. The authors reviewed relevant available documents on dengue epidemiology and conducted site visits and interviews with public health offices, health centers, referral hospitals, health insurers, and drug distribution organizations. An average of 1,543 symptomatic dengue cases was reported annually from 2011 through 2016. Intensive waste and water management on a resort island cost $1.60 per occupied room night. Local vector control programs on inhabited islands cost $35.93 for waste collection and $7.89 for household visits by community health workers per person per year. Ambulatory care for a dengue episode cost $49.87 at a health center, while inpatient episodes averaged $127.74 at a health center, $1,164.78 at a regional hospital, and $1,655.50 at a tertiary referral hospital. Overall, the cost of dengue illness in the Maldives in 2015 was $2,495,747 (0.06% of gross national income, GNI, or $6.10 per resident) plus $1,338,141 (0.03% of GNI or $3.27 per resident) for dengue surveillance. With tourism generating annual income of $898 and tax revenues of $119 per resident, results of an international analysis suggest that the risk of dengue lowers the country’s gross annual income by $110 per resident (95% confidence interval $50 to $160) and its annual tax receipts by $14 per resident (95% confidence interval $7 to $22). Many innovative vector control efforts are affordable and could decrease future costs of dengue illness in the Maldives.

## Author summary

As tourism is the mainstay of the Maldives’ economy, this country recognizes the importance of controlling mosquito-borne diseases in an environmentally responsible manner. This study sought to estimate the economic costs of dengue in this Small Island Developing State of 417,492 residents with an annual average of 1,543 reported symptomatic dengue cases. Overall, the cost of dengue illness in the Maldives in 2015 was $3 million ($6.10 per resident) and surveillance cost an additional $1 million ($3.27 per resident). The risk of dengue lowers the country’s gross annual income by $110 per resident and its annual tax receipts by $14 per resident. Rigorous elimination of debris on some resort islands demonstrates effective and environmentally sound vector control. Many innovative vector control efforts are affordable and could decrease future costs of dengue illness in the Maldives.

## Introduction

With approximately half of the world’s population at risk, dengue remains the most important mosquito-borne infection world-wide,[1, 2] costing almost $9 billion globally per year for prevention and control.[3] The ecology of Small Island Developing States and territories (SIDS), particularly with regards to temperature and precipitation, keeps dengue a continuing threat.[4] Outbreaks of dengue in SIDS can cause high burden, affecting the majority of island residents and overwhelming health systems.[5] SIDS are at risk of vector borne diseases as they are prone to natural disasters, often lack safe water supply, sanitation and waste management strategies, and their local governments have limited resources to implement effective vector control and manage outbreaks.[6]

The Republic of Maldives (the Maldives) is a South Asian SIDS in the Indian Ocean made up of around 1,192 islands. Its 417,492 residents (in 2015) lived on 187 inhabited islands (island inhabited by national residents) plus 126 resort islands, all grouped in 20 administrative atolls.[7] The capital city, Malé, is the most populous island. The Maldives ranks high in South East Asia in World Bank health indicators,[8] thanks to a well-developed public health system with a publicly funded health center on each inhabited island, a hospital for each atoll, and both public and private referral hospitals in Malé. Maldivians are enrolled in the country’s universal insurance system, Aasandha, which covers the cost of medical treatment, prescriptions, transfers, and if necessary, overseas care.

Dengue was first reported in the Maldives in 1979 and became endemic in 2004, when all atolls began reporting a high incidence of infections.[9] The Maldives’ Health Protection Agency (HPA) is responsible for dengue and vector surveillance and control, supporting personnel on each atoll and each inhabited island. As dengue is transmitted by *Aedes* mosquitoes, and in the absence of effective treatment or a public vaccination program, the main prevention strategy relies on controlling the vector population through integrated vector management.[10, 11]

Tourism is the major economic sector of the Maldives, with more than 1.2 million visitors arriving in 2014,[12] mostly from Europe. To sustain this economy, the Maldives maintains its reputation as a “paradise island destination” by ensuring a clean environment with minimal risk of infectious diseases. The coefficient (± standard error) of “dengue” in a regression on tourist arrivals was highly significant. These calculations suggest that the risk of dengue reduced the number of international tourist arrivals by 11.7% (95% confidence interval 5.3% to 18.0%) compared to the expected number without such a risk.[13]

As 9 out of 10 guests stay in self-contained resort islands, which generally practice comprehensive waste management and source reduction strategies, their risk of dengue infection is limited. Over the last decade, however, tourists increasingly visit and stay in guest houses on inhabited islands. Mosquito populations are abundant on these islands, increasing the risk of exposure to dengue virus. There are reports of tourists[14, 15] or visiting workers[16] contracting dengue in the Maldives.

Predominant vector breeding sites on inhabited islands stem from unmanaged waste and the presence of unprotected water storage containers. With support from the World Bank, the Maldives is expanding its national plan for waste management,[17] initiating a $17 million program to improve waste collection and management at regional and island levels.[18] In addition, health care workers on islands are trained to promote the safe storage of water and prevention of breeding sites to residents. Each island council is tasked with developing its own vector control strategies based on local needs to obtain national funding support. Waste management is important in the Maldives not only to mitigate the risk of *Aedes*-borne infections, but also to maintain the pristine environment underpinning the country’s tourist industry.

While the literature documents several studies on preventing and controlling dengue, [3, 19] economic studies on dengue specifically in SIDS or tourism-based economies are rare. To better understand the economic cost of dengue illness, control and preventive efforts and inform future control efforts, we undertook an economic analysis of dengue prevention and case management in this tourism-based economy.

## Methods

### Study area

The Maldives has a well-developed public health system with a publicly-funded health center on each inhabited island, a hospital for each atoll, and both public and private referral hospitals in Malé. Dengue can be treated at each of these health centers. Maldivians are also enrolled in the country’s universal insurance system, Aasandha. In addition, the State Trading Organization (STO), a public company primarily owned by the Government of the Maldives, operates at least one public pharmacy in every inhabited island where Maldivians can obtain prescribed medicines. The country’s latest (2016) gross national income per capita was US $10,630.[20]

### Study sites

**Fig 1:**
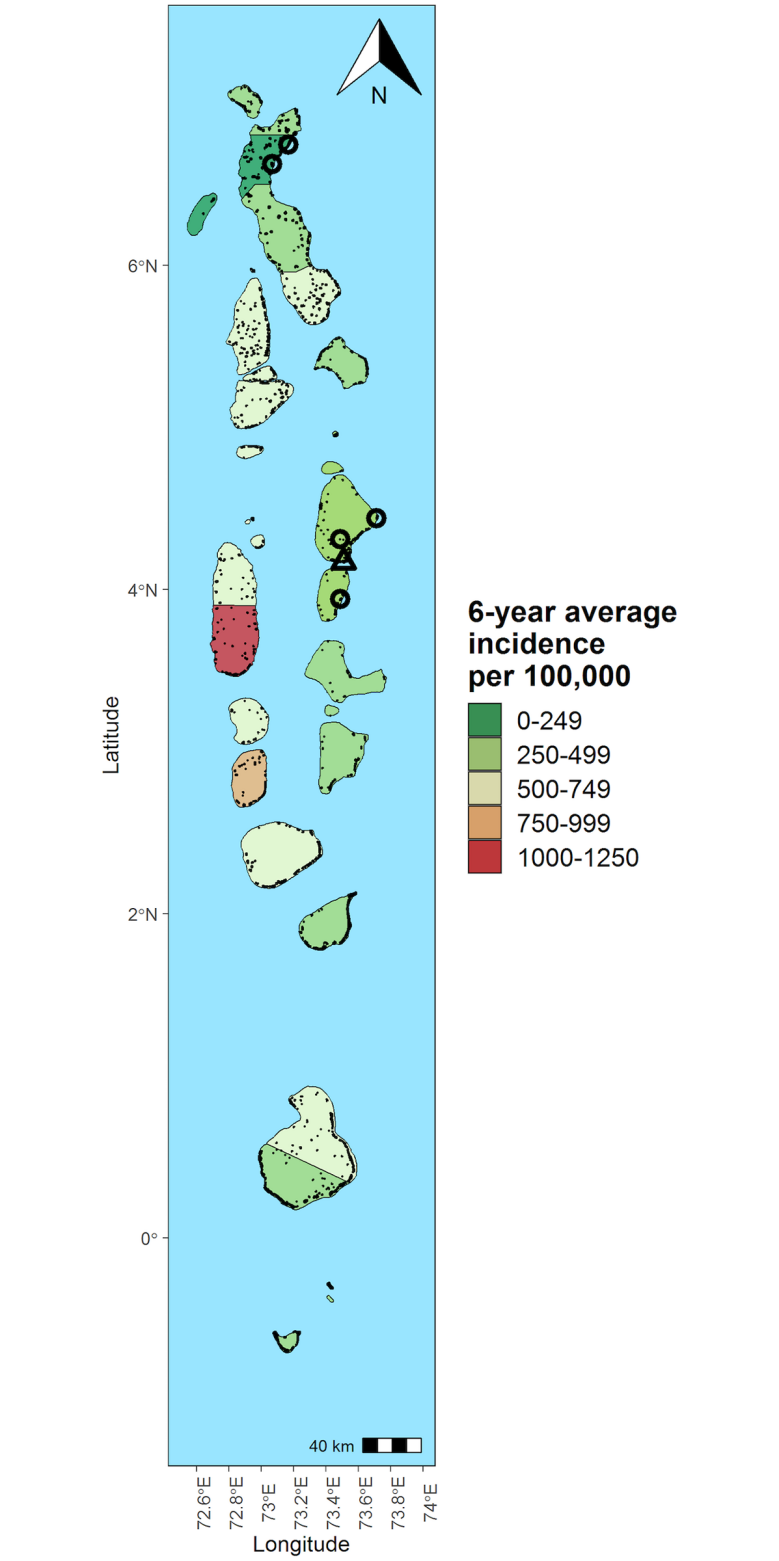
Maldives map with average dengue incidence per 100,000 population by atoll, 2011–2016. Black dots indicate islands, shading atoll boundaries, triangle Malé, and circles sites examined. GIS data by OpenStreetMap, under ODbL.Dengue incidence and risk factors.

We visited health offices and facilities across the country’s health care spectrum in December 2016. These comprised island health centers and local council offices (on Haa Dhaalu [HDh.] Hanimaadhoo, Kaafu [K.] Dhiffushi and K. Maafushi), a resort island (Thulhagiri), a regional hospital (on HDh. Kulhudhuffushi) and the Indira Gandhi Memorial Hospital (IGMH), the country’s main referral hospital (in Malé). The location of each of these sites is marked in Fig 1. We further conferred with senior officials from the HPA, National Bureau of Statistics, Allied Health Insurance, Aasandha Health Insurance, the STO, the Maldives Association of Travel and Tour Operators, Ministry of Environment and Energy, and the Ministry of Tourism.

We obtained aggregate statistical data on dengue cases from the Maldives HPA. Population data for atolls were obtained from the Maldives National Bureau of Statistics 2014 Census. The Maldives National Bureau of Statistics Housing and Household Characteristics Statistical Release 2014 provided data on waste disposal and water sources. To analyze these data, we classified atolls as “high risk” for waste disposal when they disposed of their waste “in open garbage sites” or “on the beach or in the bush”. We designated atolls as “high risk” for water storage when there was “rain water collection” or “presence of an open well.” We analyzed the relationship between risk factors related to waste disposal and water sources on dengue by regressing the six-year average incidence rate on these risk factors. To ensure comparable observations, we excluded the outlier atolls South Ari and South Thiladhunmathi as well as the capital, Malé.

### Resource inputs and costs

This study adopted a bottom-up costing approach. First, all elements of the dengue control program were identified. Thereafter, data on resource utilization and unit costs of each resource were obtained or derived for 2016. Total program costs were then derived from the sum of the product of resource utilization and unit costs for each element. Data collected included both capital and recurrent expenditures for dengue control activities. Buildings were assumed to have a 20-year useful lifespan as they were generally small, made of local materials, and faced constant sun and humidity. The one piece of equipment (thermal fogger) was assumed to have a 10-year useful life.

We recorded data for resource use and costs at the district level in a matrix by line item and function. All items combine amortized capital costs (e.g., buildings and equipment) and recurrent (e.g., utilities, fuel, and maintenance) costs. Thereafter, costs for the line items were summed up to provide the total cost of dengue control activities for each district. Data on vector control activities and scenarios about hypothetical typical cases were collected through structured interviews with managers of health centers, hospitals, island councils, and a resort. Additional financial statements and database extracts were obtained from the STO, Aasandha and IGMH. The dengue fraction reflects the supervisors’ best estimate of the share of the relevant staff time devoted to dengue control activities. All data were entered into Microsoft Excel and the statistical software R for analysis.[21]

Finally, we estimated the impact of dengue on the tourism sector using a regression coefficient from the international literature.[13] The study did not access or use any individual patient data.

## Results

### Dengue cases

**Fig 2:**
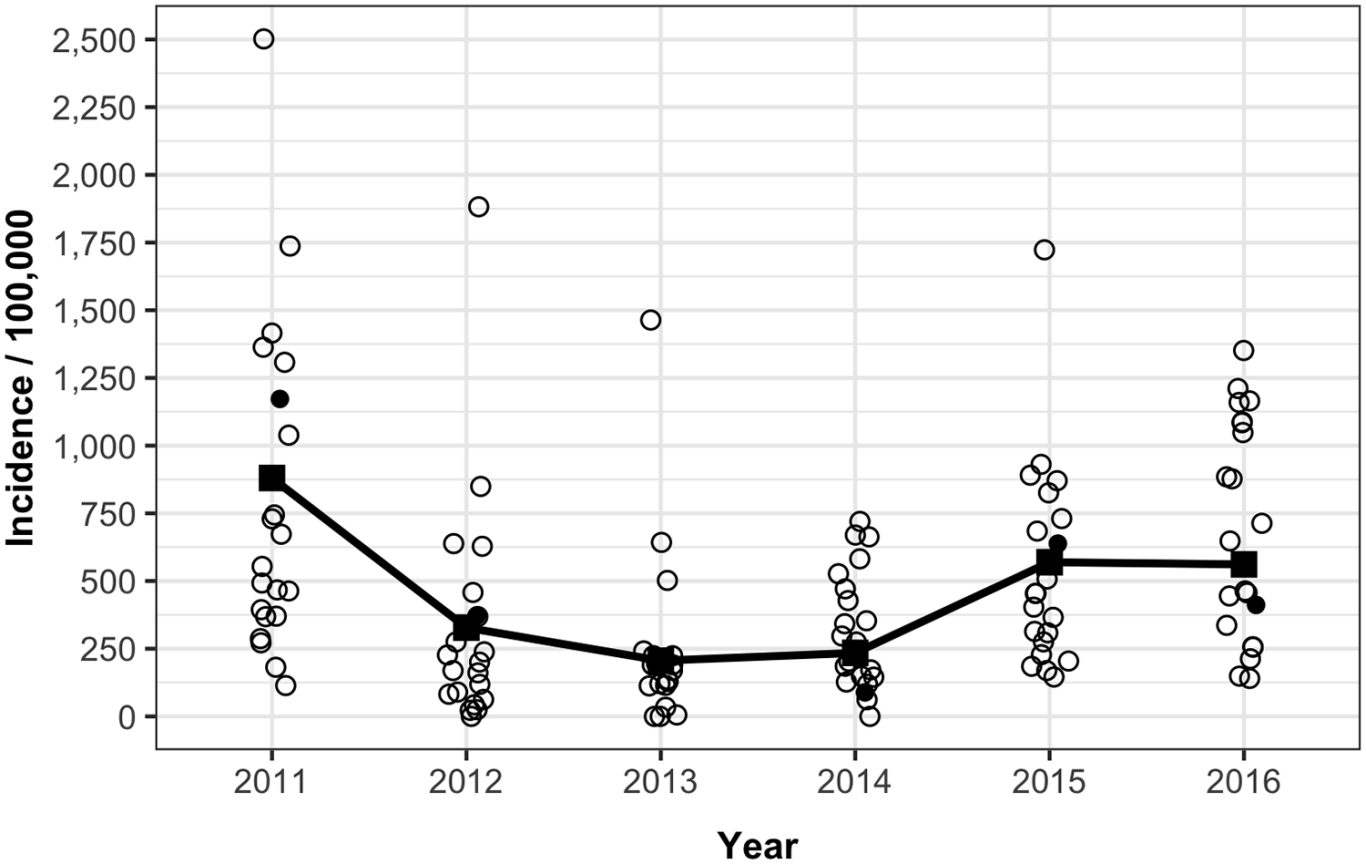
Incidence of officially reported dengue cases by atoll in the Maldives, 2011–2016. For each year, black squares depict national average dengue incidence, open circles dengue incidence for individual atolls, and the filled circle the capital.

The number of officially reported dengue cases by year from 2011 through 2016 was 2909, 1083, 680, 775, 1881, and 1931, respectively, with an annual average of 1,543 cases. Fig 2 depicts the incidence of dengue cases per 100,000 inhabitants of each atoll from 2011–2016. Based on these numbers, the average number of dengue cases per month per island health center from 2011–2016 was 0.98. The total resident population of the Maldives was 417,492 in 2016.[20]

### Dengue risk factors

**Fig 3:**
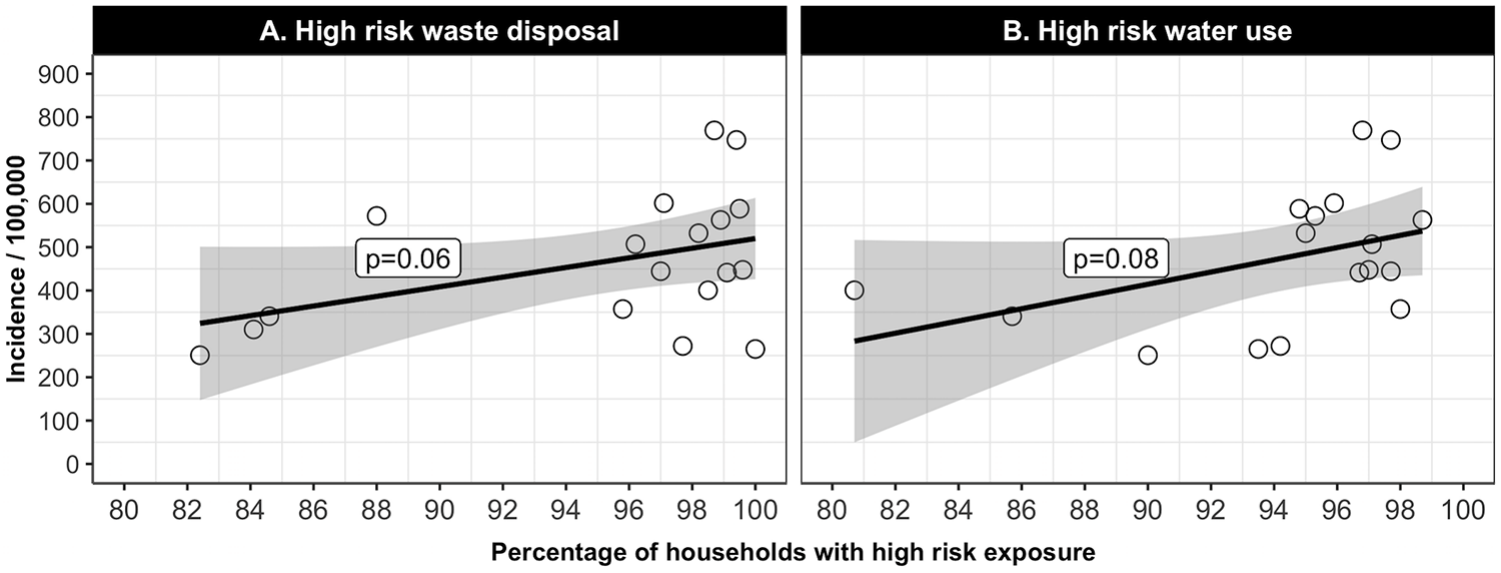
Relationship of average dengue incidence 2011–2016 to risks by atoll. High risk waste disposal: open garbage sites, the beach, or the bush. High risk water use: rain water in unsealed tank or open well. Sources: Health Protection Agency, [7].

Data from the Maldives provide suggestive evidence on the effectiveness of two source reduction strategies in controlling dengue: waste removal and water source management. Fig 3A and 3B depict dengue incidence nationally against means of waste disposal and water management, respectively. The trends suggest that for both higher percentage risky waste disposal (p = 0.06) and higher percentage risky water management (p = 0.08) are associated with elevated risk of dengue infection.

### Cost of surveillance

Table 1 shows the estimated annual cost of dengue surveillance based on staffing needs at national, regional, atoll, and island levels. The surveillance costs measured relate to the staffing and information technology required to capture, analyze and disseminate epidemiological data. The total cost for dengue surveillance is $1,338,141 per year, or $3.27 per Maldivian resident per year.

**Table 1:** Cost of dengue surveillance in the Maldives, 2015 (US$).

Level	Description		Annual Quantity	Dengue fraction	Annual unit cost	Annual cost	%
***Staff***							
National		Surveillance	2	70%	$11,439	$16,015	1%
Malé region		Surveillance & records	10	30%	$10,000	$30,000	2%
Atolls		Surveillance	40	80%	$6,667	$213,333	16%
Inhabited islands		Public health	187	60%	$6,667	$747,999	56%
***Non-personnel***							
All	Training sessions		20	100%	$15,000	$300,000	22%
All	Computers*		200	100%	$154	$30,794	2%
**Total**						**$1,338,141**	**100%**
**Cost per resident per year for population of 409,163 in 2015**^**19**^						**$3.27**	

* Capital cost of $667 annualized over 3 years with a real interest of 3% per year.

### Cost of selected vector control activities in selected islands

Local initiatives, like those on the island of Hdh. Hanimaadhoo (inhabitants: 1,800), demonstrated effective systems of source reduction–regular waste collection run by the local council. For a small fee, residents put out their waste and laborers gather it from public places. It is loaded into trucks and taken to a central place on the island for storage and, in the future, for recycling. The costs include laborers, supervision and vehicles and are described in Table 2. This initiative costs US$35.93 per island resident per year.

**Table 2:** Cost of selected vector control activities, 2015 (US$).

Description	Annual quantity	Dengue fraction	Annual unit cost	Annual cost	%
***1*. *Council waste collection on Hdh*. *Hanimaadhoo***					
Laborers	8	100%	$4,872	$38,976	60%
Drivers	2	100%	$4,872	$9,744	15%
Waste control officer (supervisor)	1	50%	$7,788	$3,894	6%
Council President (donated time)	1	25%	$7,788	$1,947	3%
Fuel and maintenance	1	100%	$4,680	$4,680	7%
Lorry ($47,000, 10 years)	1	100%	$5,448	$5,448	8%
**Total**				**$64,689**	**100%**
**Cost per person per year (population1,800)**				**$35.93**	
***2*. *Household visits on Hdh*. *Hanimaadhoo***					
Community health workers	2	25%	$7,008	$3,504	25%
Family health workers	2	25%	$7,008	$3,504	25%
Supervisor	1	25%	$7,008	$1,752	12%
Coordinator	1	100%	$5,448	$5,448	38%
**Total**				**$14,208**	**100%**
**Cost per person per year (population 1,800)**				**$7.89**	
*3*. *Fogging on K*. Dhiffushi (discontinued)*					
Insecticide (liters)	0.84	100%	$90.06	$75.65	46%
Diesel mix for insecticide (liters)	40	100%	$0.58	$23.38	14%
Labor days	6	100%	$6.82	$40.91	25%
Petrol for fogging machine (liters)	6	100%	$0.58	$3.50	2%
Machine amortization (per year)	0.1172	100%	$168.83	$19.79	12%
**Total**				**$163.23**	**100%**
**Cost per person per year (population 1,100)**				**$0.15**	
***4*. *Intensive waste and water management on a resort island***					
Staff cleaning gutters and other breeding spots	2	100%	$23,376	$46,752	67%
Staff cleaning waste on beaches, etc.	7	14%	$23,376	$23,072	33%
**Total**				**$69,824**	**100%**
Cost per occupied room night**				**$1.60**	

*Note: Fogging used to be performed twice a year for three days. Machine amortization assumes 10 year lifespan at 3% interest.

** Average of 118 guests, based on resort capacity (160) and average occupancy of the Maldives hotel resorts in 2015 (74%).[12]

A source reduction initiative, addressing household water storage and related breeding sites, involved regular household visits (approximately twice per year) to show homeowners safe water storage practices and explaining other measures to reduce breeding sites. This includes application of larvicides; *Bacillus thuringiensis* israelensis or temephos (from 2016 onwards) provision of larvivorous fish for households with wells or fresh water holding bodies and instruction about sealing the pipe connections into water tanks to prevent mosquito entry. Table 2 details the cost of these initiatives, resulting in US$7.89 per island resident per year.

The Maldives HPA currently recommends space spraying (thermal fogging or using a mist blower) as a measure only during an outbreak and in targeted priority locations. In a prior year, authorities on K. Dhiffushi conducted fogging to reduce adult mosquito populations. As shown in Table 2, fogging cost US $0.15 per year per island resident.

To reduce risks to guests and maintain the reputation of the resort as well as the country as a whole, resort islands engage in extensive vector control. The costs of these activities are described in Table 2. Thulhagiri resort employed 2 full-time staff for waste reduction, breeding site identification and gutter cleaning for the sole purpose of mosquito control for 160 guests. Another 7 employees work full time to ensure clean facilities are maintained, of which 1/7th of their time is dedicated to cleaning potential vector breeding sources. With employees’ benefits including free accommodation and food, these vector control activities cost $1.60 per hotel-guest night.

To project the cost of extending the local waste management and household visit programs nationally, we estimated that scaling could halve the per capita costs from $43.82 ($35.93 for waste collection plus $7.89 for house visits) to $21.91. The resulting national cost would be $9.1 million annually. Similarly, the projected vector control costs for resort islands would be $11.2 million annually, based on 1,234,248 tourists staying for an average of 5.7 days in 2015.[12]

### Costs of dengue cases in a health center, regional and national hospital

Table 3 first examines cost at the lowest level of the system, a health center, on the inhabited island of K. Dhiffushi. Two types of cases are considered: an ambulatory mild case of dengue, and a hospitalized case of dengue. An ambulatory case is estimated to consult the medical officer in the island health facility two times, be tested for NS1 antigen and receive a simple prescription, resulting in an overall economic cost of $49.87 per ambulatory dengue case. A hospitalized case is also estimated to consult the medical officer in the island health facility two times, be tested for NS1 antigen, receive prescriptions, and stay at the health facility for one night. This resulted in an overall economic cost of $127.74 per hospitalized dengue case. Aasandha payments for the product or service, where available, are shown as financial costs.

**Table 3:** Economic and financial costs of confirmed dengue cases in alternative settings, the Maldives, 2015 (US$).

Description	Quantity	Unit cost		Total cost	
		Financial	Economic	Financial	Economic
*Island health center on K*. *Dhiffushi*: *Ambulatory case*					
Consultations per patient (including diagnostics)	2	$6.49	$24.94	$12.99	$49.87
Medications & treatments (prescriptions)	1	$1.62	incl.	$1.62	incl.
**Total per ambulatory case**				**$14.61**	**$49.87**
*Island health center on K*. *Dhiffushi*: *Hospitalized case*					
Consultations per inpatient (including diagnostics) before and after hospitalization	2	$6.49	$24.94	$12.99	$49.88
Bed days per inpatient	1	$3.90	$77.86	$3.90	$77.86
Medications & treatments (prescriptions)	1	$14.60	incl.	$14.60	incl.
**Total per hospitalized case**				**$31.48**	**$127.74**
*Regional hospital on Hdh*. *Kulhudhuffushi*					
Consultations per inpatient (including diagnostics)	2	$0.00	$63.51	$0.00	$127.01
Bed days per inpatient	4	$0.00	$198.44	$0.00	$793.77
Medications & treatments (prescriptions)	1	$16.00	$244.00	$16.00	$244.00
**Total per hospitalized cases**				**$16.00**	**$1,164.78**
*Indira Gandhi Memorial Hospital in Malé*					
Consultations (including diagnostics)	2	n.a.	$114.17	n.a.	$228.34
Inpatient days per inpatient case	4	n.a.	$356.79	n.a.	$1,427.16
**Total per hospitalized case**				**n.a**.	**$1,655.50**

Notes: n.a. denotes not available; incl. denotes includes in economic consultation cost.

Table 3 also analyzes costs at a regional hospital. The hospital had 50 beds with 60% occupancy 365 days per year generating 10,950 bed-day equivalents. For outpatient services, it had 76,026 annual outpatient visits (about 255 per weekday), each generating 0.32 bed day equivalents,[22] or 25,480 bed day equivalents. In total, the hospital produced 36,430 bed day equivalents, of which 30% were generated by inpatient stays and 70% by outpatient visits. The estimated economic cost of one hospitalized dengue case is US$1,164.78.

Finally, Table 3 examines the economic cost of care at IGMH for 2015. Assuming an average stay for a dengue patient of 4 days, the 2015 healthcare cost of a hospitalization within IGMH was $1,655.50. The derivation of these costs is presented in Supporting Information file S1 Table.

### Payments for specialized services: Emergency evacuation

In addition to funding conventional services, Aasandha also covers certain specialized services needed because of the Maldives’ status as a SIDS with a small and dispersed population. Table 4 shows the quantities and costs of these services: sea evacuations, air evacuations, and overseas hospitalizations for 2015. With only 140 evacuations in one year, the quantity of these specialized services is low, but their aggregate cost is substantial at $116,901 in 2015. The most expensive cost was overseas hospitalization to Sri Lanka (89%) and India (11%). The average cost of the sea evacuations ($519) was almost as high as that for the air transfers ($628).

**Table 4:** Average annual payments for specialized services for dengue patients, 2015.

Description	Annual quantity	% of services	Total cost	% of costs	Cost/case
Sea transfers	140	71%	$72,722	62%	$519
Air transfers	45	23%	$28,253	24%	$628
Overseas hospitalizations	13	7%	$15,926	14%	$1,225
**Total (sea, air, overseas hospitalization)**	**198**	**100%**	**$116,901**	**100%**	**$590**

### Overall costs of dengue cases

Using epidemiological data, unit costs of hospitalization and ambulatory care from this study, and indirect costs of dengue cases infection derived from a systematic literature review,[3] we calculated the overall cost of dengue illness in the Maldives in 2015, as shown in Table 5. We estimated a total cost of $2,495,747, of which 48% was direct costs, 5% for emergency evacuations, and 47% indirect costs.

**Table 5:** Overall annual cost of dengue illness in the Maldives, 2015.

Item	Hospitalized non-fatal	Ambulatory non-fatal	Nonmedical non-fatal	Child fatal	Adult fatal	Overall
Number of cases^§^	1,141	749	1,226	2	4	3,122
Direct cost/case^#^	$999	$64	$7	$999	$999	$385
Evacuation cost/case^**+**^	$62	$62	$0	$62	$62	$37
Indir. costs/case*	$130	$73	$73	$192,000	$125,000	$377
Total cost/case	$1,190	$199	$80	$193,060	$126,060	$799
Agg. direct cost	$1,139,471	$48,296	$8,582	$1,997	$3,995	$1,196,349
Agg. evacuation cost	$70,350	$46,181	$0	$123	$247	$116,901
Agg. indir. cost	$148,330	$54,677	$89,498	$384,000	$500,000	$1,176,505
Agg. total cost	$1,358,151	$149,154	$98,080	$386,121	$504,241	$2,495,747

^**§**^Numbers of cases are based on supplementary material in Shepard et al.^3^ and estimates derived from surveillance data. They are estimated averages for three years around 2015. Such averages are often preferable to estimates for single year’s burden for diseases, such as dengue, that vary widely between successive years. Cases are classified according to the most intensive setting in which they were managed.

^#^Average direct unit costs combine unit costs from Shepard et al.^3^ and economic costs from this study adjusted for the year 2015. Direct costs of fatal cases were assumed to be the same as those for hospitalized cases. Aggregate costs of emergency transfers taken from Table 4 were pro-rated among hospitalized non-fatal, ambulatory non-fatal, and fatal cases based on numbers of cases. Even though evacuation costs are for 2015, they were the most complete available.

*Indirect costs, which were based on supplementary material in Shepard et al.^3^ and estimates from this study, reflect the economic costs of lost productivity and premature death; these are economic (non-financial) costs. Notes: Indir. denotes indirect; dir. denotes direct; agg. denotes aggregate.

The Maldives’ largest economic sector, tourism, brought a gross income of US $375 million ($898 per resident) and tax receipts of US$50 million ($119 per resident) in 2014.[12] Based on the aforementioned regression analysis,[13] the risk of dengue lowers the country’s gross annual income by $44 million ($110 per resident, 95% confidence interval $50 to $160) and its annual tax receipts by $6 million ($14 per resident, 95% confidence interval $7 to $22).

## Discussion

To our knowledge, this is the first economic evaluation of dengue prevention and control in the Maldives, a SIDS actively working to reduce the burden of vector borne diseases.[23] Using data from 2015, we found the cost of dengue in the Maldives to be $2,495,747 ($6.10 per resident) for dengue illness plus $1,338,141 ($3.27 per resident) for surveillance. The study results suggest that the economic costs through depressing tourism are substantially greater than the economic cost of illness.

This paper describes four mosquito source reduction strategies seen in the Maldives: (1) waste collection by island councils on inhabited islands, (2) household visits by community health workers in inhabited islands, (3) thermal fogging of insecticides on inhabited islands and (4) intensive waste and water management on a resort island. The costs of these interventions varied based on the setting: inhabited islands spent from $0.15 to $35.93 per person per year, while a resort island invested $1.60 per hotel guest night.

The projected cost of scaling the local waste management and household visit programs nationally was $9.1 million annually, similar to the estimated $11.2 million already spent protecting tourists on resort islands. Costs on resort islands are higher than those inhabited by regular residents due to the resort islands’ imperative to maintain a clean and safe environment for tourists, higher wages, and more comprehensive benefits for workers, while island councils on inhabited islands must allocate their resources across a range of services. Sharing best practices could likely reduce future costs compared to these projections.

Locations such as the inhabited island of Hdh. Hanimaadhoo and the resort island of Thulhagiri operate model integrated vector management programs, providing tourists staying on these islands with the best possible protection from dengue. These tourists, however, frequently visit neighboring inhabited islands, thereby increasing the risk of infection. By sharing best practices between resort islands and other inhabited islands, both tourists and locals would enjoy greater dengue protection. Regular surveillance and monitoring would allow both resort islands and the HPA to evaluate and refine their efforts. As climate change likely increases the burden of vector borne diseases,[24] extending effective vector control to all islands will become more important in the future.

Financial and economic costs of dengue case management varied depending on the location and case classification. For mild dengue cases presenting at island health centers, the cost was $49.87 per ambulatory case, and almost tripled to $127.74 for hospitalized cases. Case management of complicated dengue patients in regional or national hospitals significantly increased the cost to $1,164.78 and $1,655.50 per hospitalized case, respectively. This is more expensive than the closest neighboring island, Sri Lanka, where average costs per hospitalization are between US$196 to $866 for adult cases, depending on disease severity and treatment setting.[25]

Medical fees for non-citizens are double those for citizens, and Aasandha does not cover non-citizens. For example, health staff on K. Maafushi island estimated that foreign workers faced an incidence of symptomatic dengue 30 times that of its Maldivian residents (i.e., 300 versus 10 per 100,000 population per year). Similarly in Singapore, migrant workers are more at risk to arbovirus infection.[26] Besides a higher risk of contracting dengue, foreign workers are often reported to delay treatment, so their risk of serious illness, and possibly death, is higher. Migrant workers in the Maldives often come from dengue endemic countries and may work on several islands during their stay, thereby increasing the risk of dengue transmission throughout the country.

With its 187 inhabited islands, the Maldives necessarily relies on boats, seaplanes and airplanes for transportation. Although scheduled routes exist, medical transports frequently cannot wait and require ad-hoc emergency evacuations of patients from island health facilities to referral hospitals, including chartering a speed boat. In our study we found that despite a high number of evacuations, these contributed to only 7% of total costs. We found plane evacuations more economic than boats, likely due to difference in costs of purchasing a plane ticket on scheduled flights compared to the cost of chartering a speed boat. Hospitalizations of complicated dengue cases overseas in our study cost on average $1,225. This is in line with the previously reported average cost of $1,470 to obtain treatment of any kind in overseas health facilities from the Maldives.[27] The aggregate cost of overseas hospitalizations for dengue in 2015 ($15,926), however, forms only a small part of the estimated $68.9 million spent on overseas hospitalization for all conditions for Maldivian citizens.[27]

We estimated that each island health facility clinician sees on average of only one dengue case per month. Due to this low frequency and high turnover of clinicians on these islands, investing resources in strengthening clinical capacity for dengue would be relatively ineffective. Maldivian authorities have implemented a “dengue hotline” to allow inexperienced clinicians to seek advice from experts at IGMH. Similar health hotlines exist in the UK[28, 29] and the USA,[30] which contribute to the triage and surveillance of cases. The current limitation of the hotline in the Maldives, however, is that calls are not screened, and island clinicians use the service for complicated dengue cases only. An expanded service could advise callers on interpretation of symptoms and test results to assess the likelihood of dengue, appropriate treatment to minimize risk, and decide appropriately on transfers. Based on international experience, we expect that an enhanced hotline would prove favorable in terms of better patient outcomes, less disruption to patients and families, and lower costs to the Maldivian health care system. We suggest that the Ministry of Health consider introducing an enhanced hotline phased in by atolls covered, monitoring hotline calls under both existing and enhanced phases to examine utilization, health outcomes, and costs of services and evacuations, and extending and refining the enhanced hotline based on lessons learned.

This study is limited in that only a selected number of interventions were costed, and as such, may not reflect the costs seen on each island. Furthermore, this study did not address the burden of other important arboviruses, such as chikungunya and Zika, which also have been reported in the Maldives.[31, 32] Including this in the analysis would likely increase the cost of case management, but not the cost of vector control activities, as the diseases share the same mosquito vector. In estimating the total cost of dengue in the Maldives, some of the data items were not available for the country and had to be derived from results in other countries. Using country-specific data for those items, if available, would strengthen the analysis.

Based on the importance of the tourism sector the need to mitigate the potential impact of climate change, the Maldives introduced a “green tax,” charging tourists on resort islands and guest houses $6 and $3 per person per night, respectively. While this tax revenue does not directly support the health sector, it is levied to strengthen solid waste management throughout the Maldives. By reducing plastic and other receptacles that are used by *Aedes* mosquitos to breed, this tax indirectly lowers the burden of dengue. Some additional transport or tourism tax could be established, if necessary, to fund the time, travel, and follow-up expenses needed to expand vector control services nationally. This study reinforces the economic rationale for investment in effective dengue control.

## Supporting information

S1 Table Macro costing of IGMH national hospital for 2014 and 2015, and Hdh. Kulhudhuffushi Regional Hospital for 2015.
(DOC)

## References

[1] Bangert M, Molyneux DH, Lindsay SW, Fitzpatrick C, Engels D. The cross-cutting contribution of the end of neglected tropical diseases to the sustainable development goals. Infect Dis Poverty. 2017;6(1):73 10.1186/s40249-017-0288-0 28372566 PMC5379574

[2] World Health Organization. Dengue and Severe Dengue 2017. http://www.who.int/mediacentre/factsheets/fs117/en/. Cited 17 Aug 2017.

[3] Shepard DS, Undurraga EA, Halasa YA, Stanaway JD. The global economic burden of dengue: a systematic analysis. Lancet Infect Dis. 2016;16(8):935–941. 10.1016/S1473-3099(16)00146-8 27091092

[4] Feldstein LR, Brownstein JS, Brady OJ, Hay SI, Johansson MA. Dengue on islands: A Bayesian approach to understanding the global ecology of dengue viruses. Trans R Soc Trop Med Hyg. 2015;109(5):303–312. 10.1093/trstmh/trv012 25771261 PMC4401210

[5] Emerging Disease Surveillance and Response Team, Division of Health Security and Emergencies, World Health Organization Regional Office for the Western Pacific, Arima Y, Chiew M, Matsui T. Epidemiological update on the dengue situation in the Western Pacific Region, 2012. West Pac Surveill Response J. 2015;6(2):82–89. 10.5365/WPSAR.2014.5.4.002 PMC4542491 26306221

[6] Cao-Lormeau V-M. Tropical islands as new hubs for emerging arboviruses. Emerg Infect Dis. 2016;22(5):913–915. 10.3201/eid2205.150547 27088243 PMC4861506

[7] Maldives Ministry of Finance and Treasury, National Bureau of Statistics. Statistical Release I: Population and Households 2014. http://statisticsmaldives.gov.mv/statistical-releases/. Cited 16 January 2018.

[8] The World Bank. World Development Indicators 2017. http://wdi.worldbank.org/table/2.12. Cited 16 August 2017.

[9] Abdulla AA, Rasheeda F, Ahmed IN, Aboobakur M. An evaluation of the surveillance system for dengue virus infections in Maldives. WHO South-East Asia J Public Health. 2014;3(1):60–68. 10.4103/2224-3151.206886 28607256

[10] Fitzpatrick C, Haines A, Bangert M, Farlow A, Hemingway J, Velayudhan R. An economic evaluation of vector control in the age of a dengue vaccine. PLoS Negl Trop Dis. 2017;11(8):e0005785 10.1371/journal.pntd.0005785 28806786 PMC5573582

[11] World Health Organization. Handbook for Integrated Vector Management. Geneva: World Health Organization; 2012. http://apps.who.int/iris/bitstream/10665/44768/1/9789241502801_eng.pdf?ua=1. Cited 4 August 2018.

[12] Maldives Ministry of Tourism. Tourism Yearbook 2016. http://www.tourism.gov.mv/?wpdmdl=10490. Cited 16 August 2017.

[13] Rosselló J, Santana-Gallego M, Awan W. Infectious disease risk and international tourism demand. Health Policy Plan. 2017;32:538–548. 10.1093/heapol/czw177 28104695

[14] Napoli C, Salcuni P, Pompa MG, Declich S, Rizzo C. Estimated imported infections of chikungunya and dengue in Italy, 2008 to 2011. J Travel Med. 2012;19(5):294–297. 10.1111/j.1708-8305.2012.00640.x 22943269

[15] Savini H, Gautret P, Gaudart J, Field V, Castelli F, López-Vélez R, et al Travel-associated diseases, Indian Ocean Islands, 1997–2010. Emerg Infect Dis. 2013;19(8):1297–1301. 10.3201/eid1908.121739 23876977 PMC3739505

[16] Trojánek M, Tomíčková D, Roháčová H, Kosina P, Gebouský J, Dvořák J, et al [Dengue fever cases in Czech workers returning from the Maldives]. Epidemiol Mikrobiol Imunol. 2013;62(3):100–105. 24116697

[17] Environment Research Centre, Ministry of Environment Energy and Water. National Solid Waste Management Policy for the Republic of Maldives 2008. http://www.mvlaw.gov.mv/pdf/gavaid/minHousing/28.pdf. Cited 26 January 2018.

[18] The World Bank. Projects & Operations, Maldives Clean Environment Project 2018. http://projects.worldbank.org/P160739/?lang=en&tab=overview. Cited 26 January 2018.

[19] Stahl H-C, Butenschoen VM, Tran HT, Gozzer E, Skewes R, Mahendradhata Y, et al Cost of dengue outbreaks: Literature review and country case studies. BMC Public Health. 2013;13(1):1. 24195519 10.1186/1471-2458-13-1048 PMC4228321

[20] The World Bank. Country Data, Maldives 2018. https://data.worldbank.org/country/maldives. Cited 30 January 2018.

[21] R Core Team. R: A Language and Environment for Statistical Computing. Vienna, Austria: R Foundation for Statistical Computing 2016. https://www.R-project.org/. Cited 26 January 2018.

[22] Shepard DS, Hodgkin D, Anthony YE. Analysis of Hospital Costs: A Manual for Managers. Geneva: World Health Organization; 2000.

[23] Ibrahim HEAN, Mathur A. Climate change and health in Maldives: Protecting our common future. WHO South-East Asia J Public Health. 2017;6(2):1–2. 28857055 10.4103/2224-3151.213785

[24] Liu-Helmersson J, Quam M, Wilder-Smith A, Stenlund H, Ebi K, Massad E, et al Climate change and Aedes vectors: 21st century projections for dengue transmission in Europe. EBioMedicine. 2016;7:267–277. 10.1016/j.ebiom.2016.03.046 27322480 PMC4909611

[25] Thalagala N, Tissera H, Palihawadana P, Amarasinghe A, Ambagahawita A, Wilder-Smith A, et al Costs of dengue control activities and hospitalizations in the public health sector during an epidemic year in urban Sri Lanka. PLoS Negl Trop Dis. 2016;10(2):e0004466 10.1371/journal.pntd.0004466 26910907 PMC4766086

[26] Sadarangani SP, Lim PL, Vasoo S. Infectious diseases and migrant worker health in Singapore: A receiving country’s perspective. J Travel Med. 2017;24:4. 10.1093/jtm/tax014 28426114

[27] Suzana M, Mills A, Tangcharoensathien V, Chongsuvivatwong V. The economic burden of overseas medical treatment: A cross sectional study of Maldivian medical travelers. BMC Health Serv Res. 2015;15:418 10.1186/s12913-015-1054-2 26409472 PMC4583732

[28] Cooper DL, Smith G, Baker M, Chinemana F, Verlander N, Gerard E, et al National symptom surveillance using calls to a telephone health advice service—United Kingdom, December 2001-February 2003. MMWR Suppl. 2004; 53:179–183. 15717389

[29] Doroshenko A, Cooper D, Smith G, Gerard E, Chinemana F, Verlander N, et al Evaluation of syndromic surveillance based on National Health Service Direct derived data—England and Wales. MMWR Suppl. 2005;54:117–122. 16177702

[30] Yih WK, Teates KS, Abrams A, Kleinman K, Kulldorff M, Pinner R, et al Telephone triage service data for detection of influenza-like illness. PLoS ONE. 2009; 4(4):e5260 10.1371/journal.pone.0005260 19381342 PMC2668187

[31] Korhonen EM, Huhtamo E, Smura T, Kallio-Kokko H, Raassina M, Vapalahti O. Zika virus infection in a traveller returning from the Maldives, June 2015. Euro Surveill. 2016;21(2). 10.2807/1560-7917.ES.2016.21.2.30107 26794427

[32] Yoosuf AA, Shiham I, Mohamed AJ, Ali G, Luna JM, Pandav R, et al First report of chikungunya from the Maldives. Trans R Soc Trop Med Hyg. 2009;103(2):192–196. 10.1016/j.trstmh.2008.09.006 18930301

