# Supplementary material for: Economic analysis of dengue prevention and case management in the Maldives
Source: PLoS Negl Trop Dis. 2018 Sep 27;12(9):e0006796. doi: 10.1371/journal.pntd.0006796 (PMC6177194; doi:10.1371/journal.pntd.0006796)
Supplement: S1 Table — Kulhudhuffushi Regional Hospital for 2015. (DOC) [file pntd.0006796.s001.doc]

Table S1. Macro costing of IGMH national hospital for 2014 and 2015, and Hdh. Kulhudhuffushi Regional Hospital for 2015

|  | **IGMH** | | **Hdh. Kulhudhuffushi** |
| --- | --- | --- | --- |
| **Item** | **2014** | **2015** | **2015** |
| Number of registered beds (official) | 275 | 275 | 50 |
| Occupancy rate | 74% | 73% | 60% |
| Average number of patients per night | 204 | 199 | 30 |
| Annual bed days | 74,468 | 72,812 | 10,950 |
| Total ambulatory and preventive care visits | 311,830 | 333,585 | 79,560 |
| Relative cost: visit/inpatient day[22] | 0.32 | 0.32 | 0.32 |
| Ambulatory bed-day equivalents | 99,786 | 106,747 | 25,459 |
| Total bed day equivalents | 174,254 | 179,559 | 36,409 |
| Operating expenditure | $36,180,796 | $51,231,931 | $ 5,974,026 |
| Drugs supplied by STO | $11,755,716 | $12,833,699 | $ 1,250,000 |
| Total costs of patient care | $47,936,512 | $64,065,630 | $ 7,224,026 |
| Cost per bed-day equivalent | $ 275.10 | $ 356.79 | $ 198.41 |
| Cost per visit | $ 88.03 | $ 114.17 | $ 63.49 |
| Per capita GNI of the Maldives[20] | $ 9,630.00 | $ 9,880.00 | $ 9,880.00 |
| Hospital bed day as % of per capita GNI | 2.9% | 3.6% | 0.1% |

Notes: IGMH denotes Indira Gandhi Memorial Hospital; STO denotes State Trading Organization; GNI denotes gross national income. Monetary amounts are in US dollars for each year.
